# The Unprotected: Measles Seroprevalence in Children During the First Two Years of Life

**DOI:** 10.3390/v17070973

**Published:** 2025-07-11

**Authors:** Sophie Rettenbacher-Riefler, Ina Holle, Mareike Wollenweber

**Affiliations:** 1 Department for Microbiology, Public Health Agency of Lower Saxony, 30449 Hanover, Germany; 2 Department for Infectious Disease Epidemiology and Surveillance, Public Health Agency of Lower Saxony, 30449 Hanover, Germany

**Keywords:** measles, eradication, seroprevalence, maternal antibodies

## Abstract

Young children are particularly vulnerable to measles infections. Investigating the gap between the waning of maternal antibodies and onset of vaccination-induced immunity via seroprevalence studies can be hampered by recruiting enough young-aged participants. We present measles IgG-antibody results from 2148 patients aged 0 to 2 years, who were hospitalized with acute aseptic meningitis or encephalitis in Lower Saxony or Bremen. Measles serology was performed for differential diagnostics clarification of neurotropic pathogens, during syndromic surveillance between 2006 and 2024. At birth, 79% of children presented with measles IgG-antibodies, but only 30% of three-month-old patients and 11% of five-month-olds. From 0 to 10 months, seropositivity declined monthly by 8%. Over 95% of children aged six to 11 months were unprotected. From 11 months onwards, measles seroprevalence increased, reaching 80–90% towards the end of the second year of life. Our results indicate an absence of maternal measles IgG antibodies after nine months of age and that vaccination starts around 11 months of age; however, not all children had received vaccination by their second birthday. These findings confirm the current recommendation to advance first measles vaccination to nine months in high-exposure settings and support efforts to increase vaccination rates in small children and young adults.

## 1. Introduction

Despite considerable efforts, measles is not yet eradicated. In Germany, the highest age-specific incidences are regularly observed in children during their first two years of life [1]. Complications also occur more frequently in young children [2]. Between 2001 and 2025, the German federal state of Lower Saxony registered 2493 measles cases. Children aged 0–4 years and children aged 5–9 years each accounted for 27% of cases, respectively. Considering reported outbreaks, children aged 0 to 9 years and 10 to 19 years represented 53% and 33% of all outbreak cases, respectively [3]. There is no specific antiviral therapy for the treatment of measles, and disease control largely depends on prevention, with vaccination as the most efficient measure [2]. In order to develop appropriate recommendations for vaccine-preventable diseases, seroprevalence data as well as vaccination coverage rates must be considered [4,5]. For effective transmission interruption, 95% of a population are required to have immunity against measles [1,6], acquired either by vaccination or through infection with wild-type measles virus. For targeted public health actions, identifying critical immunity gaps in populations is therefore essential. Retrospective serosurveys are considered an inexpensive and useful approach to identify susceptible populations; however, young children are often underrepresented [7], and children under 24 months of age are often not considered. Ample research over the last decades has shown that the duration of measles protection via maternal antibodies is shorter in children whose mothers had received measles vaccination, compared to children whose mothers had obtained immunity via measles infection [8,9]. Consequently, an increasing percentage of infants are no longer sufficiently protected before receiving the first dose of measles vaccination, and advancement of measles vaccination has been suggested [10]. In Germany, the recommended period for the administration of the first measles vaccination (MMR, combined with mumps and rubella) was shifted from 12 to 15 months of age to 11 to 14 months of age in 2001. In the same year, recommendation for the second MMR vaccination was advanced from 5 to 6 years of age to 15 to 23 months [11]. In 2020, the time window for the first and second MMR vaccinations was shortened and brought forward to the 11th and 15th month of life [12]. Nevertheless, these measures are most likely not sufficient to compensate for the time period of measles susceptibility between maternal antibody decay and onset of immunization. More knowledge about the seroprevalence in young children is therefore needed. Seroprevalences for children in Germany during their first two years of life have not been assessed in detail recently, and recruiting sufficient study participants in this young age group might be challenging. We therefore used laboratory results from a patient collective to calculate seroprevalences for different age groups, with a detailed evaluation focusing on children aged two years or younger. Patients were examined in the context of a syndromic surveillance system called “Meningitis and Encephalitis register in Lower Saxony (MERIN)”.

MERIN was implemented in the German federal state of Lower Saxony in 2003 and extended to the federal state of Bremen in 2011. Hospitals and clinics with pediatric, neurologic and internal medicine wards submit samples from patients with acute aseptic meningitis, encephalitis or polio-like symptoms such as acute flaccid paralysis to the virological laboratory of the Public Health Agency of Lower Saxony (NLGA) for diagnostic clarification. Different samples are analyzed using various diagnostic methods for a range of neurotropic pathogens. Serological assessment of anti-measles antibodies is part of the diagnostic differentiation for neurotropic pathogens causing aseptic meningitis, encephalitis or polio-like symptoms. Individual laboratory results are reported back to treating physicians to facilitate medical therapy. Aggregated data on circulating pathogens are analyzed at the NLGA and results on enteroviruses are forwarded to the National enterovirus surveillance to ultimately document the polio-free status of Germany [13]. High acceptance of the surveillance among stakeholders, as well as the timeliness and completeness of MERIN data, have been assessed previously [14]. The main contributors to this voluntary passive syndromic surveillance are pediatric clinics and hospital wards; therefore, the majority of MERIN patients investigated in the context of MERIN are children, predominantly children under ten years of age [13]. We analyzed anti-measles antibody results from the MERIN patient collective to assess measles seroprevalences, focusing on children during their first two years of life.

## 2. Materials and Methods

### 2.1. Serological Analysis

Serum or blood samples arriving at the laboratory of NLGA were analyzed for anti-measles immunoglobulin G antibodies (measles IgG), using a commercially available enzyme-linked immunosorbent assay, according to manufacturer’s instructions (Virotech Diagnostics Ltd., Dietzenbach, Germany). The results were rated as positive, negative, or intermediate. Based on a WHO standard reference, seropositivity corresponds to IgG concentrations protective against measles [15]. According to the manufacturer, sensitivity and specificity were 98.6% and 99.8%, respectively. The NLGA laboratory has been accredited since 2005 by the German accreditation body, and all laboratory procedures including sample management, analyses and data processing are performed in accordance with standard procedures. Laboratory results and anonymized personal data of patients are transferred weekly from the laboratory information management system to a separate MERIN database (Access, Microsoft, Redmond, WA, USA).

### 2.2. Data Analyses

Data from MERIN patients were anonymized and extracted from the MERIN database, comprising information on measles IgG results, sex, month and year of birth, as well as arrival date of the sample at the laboratory. The latter was used to determine patients’ age at the time of investigation by calculating the interval between birth date and arrival date. The present analysis comprises data from MERIN patients that were investigated from the year 2006 to 2024. Data from 2003 to 2005 were excluded, as patient numbers were low and measles IgG measurements were not performed frequently during the initial years of the surveillance. Patient data were stratified based on patients’ age in years, so that, for example, age group 1–2 represents patients who were in their first or second year of life at the time of investigation (Table 1). In addition, data from patients until 24 months of age were stratified into age groups by their age in months, so that age group 0 represents all patients that were investigated in the same month as they were born, and so that age group 1 month represents all patients that were investigated in the month after they were born (Table 2). For each age group, seroprevalence was calculated as the number of patients with positive measles IgG results divided by the total number of patients investigated in the respective age group together, with 95% Clopper–Pearson exact confidence intervals (95% CI). Piecewise linear regression was performed to investigate the monthly changes in seroprevalence for patients between 0 and 10 months of age. To determine whether measles seropositivity during the first ten months of life was associated with age, sex or birth year of patients, these variables were tested via a logistic regression model, applying a backwards selection process with a significance level of *p* < 0.05.

## 3. Results

Information on measles serostatus was available for 9626 patients with symptoms of aseptic meningitis, encephalitis or polio-like symptoms, who were hospitalized in Lower Saxony or Bremen and investigated within the framework of MERIN from 2006 to 2024. Age could be calculated for 9621 patients, and information on sex was missing in 101 patients. The overall seroprevalence was 78% (95% CI 77.2–78.9), with the lowest seroprevalence at 36.4% (95% CI 33.9–38.9) in children under 1 year of age, and the second lowest in children in their first and second year of life (80.6%, 95% CI 78.3–82.9). The second highest seroprevalence was found in patients aged 40–59 years (97.4%, 95% CI 94.0–99.1) and the highest seroprevalence was found in patients who were 60 years and older (98.5%, 95% CI 95.6–99.7) at the time of investigation (Table 1). Information on measles antibody status was available for 2148 children aged from 0 to 24 months, with an overall seroprevalence of 49.9% (95% CI 47.8–52.0), but seroprevalences differed greatly between age groups (Table 2).

**Figure 1:**
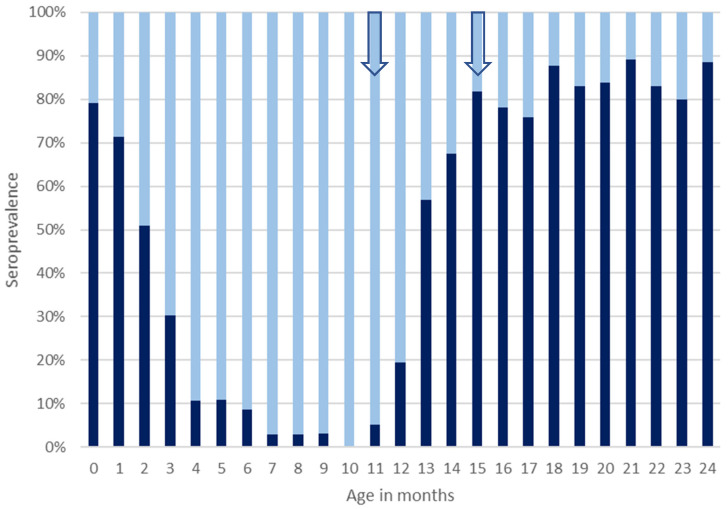
Measles seroprevalence in MERIN (Meningitis and Encephalitis Registry) patients aged 0 to 2 years from Lower Saxony and Bremen, Germany, investigated between 2006 and 2024. Dark bars represent percentages of patients with protective levels of anti-measles IgG antibodies (seropositivity) in the respective age group, and light bars represent percentages of patients with negative or intermediate concentrations of anti-measles IgG antibody levels that are considered non-protective (seronegativity). Arrows indicate the onset of the recommended age periods for first and second measles vaccination.

Around the time of birth, a seroprevalence of 79.1% (95% CI 72.9–85.4) was found, whereas in patients aged 3 months, seroprevalence was 30.2% (95% CI 21.5–39.0), and in 7-month-old patients, seroprevalence was only 2.8% (95% CI 0.3–9.8). In patients between 7 and 11 months of age, seropositivity remained below 5%, and a seroprevalence of 0% (95% CI 0.0–4.2) was found in the group of patients investigated at 10 months of age. Seropositivity increased from the age group of 11 months onwards, and a seroprevalence of 81.8% (95% CI 72.5–91.1) was observed in patients at 15 months of age. In patients aged between 16 and 24 months at time of investigation, seroprevalence levels ranged from 75.9% (95% CI 64.8–86.9) at 17 months of age to 89.1% (95% CI 77.7–95.9) at 21 months of age (Figure 1). Piecewise linear regression revealed that between month 0 and 10, the levels of seropositivity declined each month by 8% points to a minimum of 0% at the age of 10 months. Logistic regression revealed a significant association between age in months and seropositivity (OR = 0.48; 95% CI 0.44–0.53; *p* < 0.0001) during the first ten months of life, whereas sex was not significantly associated with seropositivity. The birth year of patients aged up to two years affected seropositivity, with OR = 0.96 (95% CI 0.93–0.99; *p* = 0.017) for each year born later.

## 4. Discussion

The present paper presents data from a collective of patients who were hospitalized with symptoms of aseptic meningitis, encephalitis or polio-like symptoms in clinics and hospitals in Lower Saxony and Bremen within the MERIN framework in the years 2006 to 2024. The majority of patients were children, and measles IgG levels were quantified as part of the differential diagnostic clarification for neurotropic pathogens. We calculated measles seroprevalences in different age groups to assess potential gaps in immunity, in particular during the first two years of life. The overall seroprevalence was 78%, and thus measles susceptibility was far above WHO recommendations [1]. Seroprevalence, however, differed greatly between age groups, with the highest seroprevalence found in patients who were 40 years and older at the time of investigation. Similar observations have been made in various studies across different countries, demonstrating a decline in both seroprevalence and antibody concentrations in adults in relation to the introduction of measles vaccination [7,16,17]. Older age cohorts most likely acquired immunity by exposure to wild-type measles virus, which resulted in higher concentrations of measles IgG antibodies as well as seroprevalences that meet the recommended population immunity of 92–94% [18]. The increase in seroprevalence by 10%, from 87.4% in the age group of 20 to 39-year-olds to 97.4% in the age group of 40 to 59-year-olds, might to some extent reflect this situation in our data. In children, the highest seroprevalence was found in patients that were investigated in their third and fourth year of life. Seroprevalence declined from 92.2% in this age group to 82.7% in the 10–14-years age group. Aside from patients in their first and second year of life, seroprevalence was lowest in patients investigated between the ages of 10 and 19 years, an age cohort that constitutes 33% of all outbreak cases in Lower Saxony [3]. Monitoring seroprevalence as well as making efforts to increase vaccination rates are particularly important for both outbreak prevention and the protection of infants, as these generations will become parents in the future. Mothers’ measles antibody IgG concentrations are mirrored in the infants’ antibody concentrations, and babies who were seronegative at birth were born from seronegative mothers [19]. In our patient collective, the seroprevalence of 79.1% at 0 months of age indicates that roughly a mere four out of five newborns were sufficiently protected against measles by maternal antibodies, similarly to findings in a recent study [16]. The duration of protection by maternal antibodies is determined by the amount of transferred antibodies, and faster antibody decay in the infants of vaccinated mothers is consistently described across the literature [8], resulting in children being unprotected from around 4 to 6 months of age onwards [9,16,19,20,21,22]. Our findings are in line with these reports. Comparing different age groups, we observed a rapid decline in seropositivity during the first ten months of life, as over 90% of patients were seronegative from 6 to 11 months of life, and 95% were seronegative from 7 to 10 months of life. Protective levels of measles antibodies were not detected in any of the 86 patients aged 10 months at the time of investigation. This decrease is reflected in the regression analysis, with a highly significant odds ratio of 0.48 for age in months. Differences between male and female patients were not observed. The association between year of birth and seroprevalence could reflect the decreasing proportion of children born from mothers with immunity acquired from infections with wild-type measles virus. Although we cannot differentiate between maternal measles antibodies and antibodies produced in response to vaccination or infection, our findings strongly suggest that after nine months of age, maternal measles IgG antibodies were no longer present in our patient collective. The observed seroprevalence of 19.4% in patients investigated in their 12th month of life likely indicates that vaccination starts around the recommended time point for the first MMR vaccination at 11 months of age [11,12] and that the majority of patients must have received at least one dosage at the end of their second year of life. However, one out of five children was still unprotected at this age. Vaccination rates of 92–96% at 24 months of age were recently reported [23]. The finding that the increase in seroprevalence during the second half of the second year of life continued into the third and fourth year of life could suggest that a large proportion of patients received the second MMR vaccination after their second birthday. It has been described that about half of all children in Germany are vaccinated later than recommended [24] and that measles vaccinations are still given up until school age [1,23]. Continuation of the recently described increase in vaccination rates [25] would be desirable, and further seroprevalence studies should also monitor the effect of the measles protection act introduced in 2020 on the population’s seroprevalence in Germany, especially in children [26]. Retrospective serosurveys are considered an inexpensive and useful approach to identifying susceptible populations, especially groups that are hard to recruit otherwise [7]. We therefore decided to analyze measles IgG results from our MERIN patient collective, focusing on patients that were investigated during their first two years of life. Hospitals and clinics voluntarily participate in MERIN, and it was found that the majority of their eligible patients are submitted to this syndromic surveillance [14]; therefore, influences from socio-economic status or geographic location on patients’ selection were not expected. Due to the recruitment procedure, selection bias caused by language barriers, attitudes towards vaccination or willingness to participate in scientific studies were also not likely. Infections with pathogens that cannot be prevented by vaccinations, such as enterovirus, adenovirus and Borrelia spp., constitute the majority of diagnoses within MERIN [13]. Due to the specific set-up of MERIN, a claim for the representativeness of our dataset for the whole population of Lower Saxony and Bremen cannot be made. Another limitation is that our qualitative results do not allow for conclusions about antibody concentrations. A decrease over time within the same individual also cannot be derived from the single point measurements. The findings from this secondary data analysis should certainly be researched in depth via specifically designed follow-up investigations. Regarding future seroprevalence studies, our small-scale representation could provide guidance on selecting the most suitable time points for sampling, thereby minimizing the number of blood samples collected from infants, which might increase parental compliance. Overall, the observed gap in measles susceptibility between maternal antibody decay and the onset of immunization in this patient collective from two German federal states must be taken into consideration. According to our results, over 90% of children do not have protective levels of measles antibodies for about half a year during their first year of life. This underlines the necessity to protect this vulnerable group with strong herd immunity and to have vigorous surveillance and outbreak management systems in place. This approach is preferable to further advancing immunization time points [21] because vaccine effectiveness and antibody titers are higher when measles vaccination is administered later in life [2]. Each country should, however, consider adapting the timing of measles vaccination in relation to its sero-epidemiological situation [8], balancing the need to reduce the numbers of measles-susceptible infants against the risk of compromising vaccine efficacy [16]. Increasing measles immunity in young adults has been suggested [16,18], and our findings support this necessity, particularly in view of the fact that MMR vaccination is contraindicated during pregnancy [2,12]. Our findings also underline the urgent need for catch-up vaccinations in school children to close immunity gaps [5].

## 5. Conclusions

In our study, we detected a prolonged time period in measles susceptibility between maternal antibody decay and the onset of immunization, despite advancing the first measles vaccination to 11 months of age. According to our results, maternal measles IgG-antibodies are no longer present after nine months of age, and vaccination starts around 11 months of age; however, not all children are vaccinated by their second birthday. Our findings confirm the current recommendation to advance the first measles vaccination to nine months in high-exposure settings and support efforts to increase vaccination rates in small children, as well as also young adults, who will eventually become parents and primary caregivers of young children.

## Figures and Tables

**Table 1:** Measles IgG antibody results of MERIN (Meningitis and Encephalitis Registry) patients investigated between 2006 and 2024 in Lower Saxony and Bremen, Germany, stratified by age.

Age Group (Year of Life)	Measles IgG			Total	Seroprevalence [%]	95% CI [%]
	Positive	Intermediate	Negative			
<1	511	71	822	1404	36.4%	33.9–38.9
1–2	947	16	212	1175	80.6%	78.3–82.9
3–4	806	15	53	874	92.2%	90.4–94.0
5–9	1791	72	205	2068	86.6%	85.1–88.1
10–14	1681	85	266	2032	82.7%	81.1–84.4
15–19	1224	77	184	1485	82.4%	80.5–84.4
20–39	173	6	19	198	87.4%	82.7–92.0
40–59	186	0	5	191	97.4%	94.0–99.1
60+	191	2	1	194	98.5%	95.6–99.7
missing	3	0	2	5		
Total	7513	344	1769	9626	78.0%	77.2–78.9

**Table 2:** Measles IgG antibody results of MERIN (Meningitis and Encephalitis Registry) patients up to 2 years of age, investigated between 2006 and 2024 in Lower Saxony and Bremen, Germany, stratified by age.

Age Group (Month of Life)	Measles IgG			Total	Seroprevalence [%]	95% CI [%]
	Positive	Intermediate	Negative			
0	129	9	25	163	79.1%	72.9–85.4
1	219	19	69	307	71.3%	66.3–76.4
2	89	17	69	175	50.9%	43.5–58.3
3	32	5	69	106	30.2%	21.5–39.0
4	11	6	87	104	10.6%	4.7–16.5
5	9	6	68	83	10.8%	5.1–19.6
6	8	3	81	92	8.7%	3.8–16.4
7	2	1	68	71	2.8%	0.3–9.8
8	2	2	66	70	2.9%	0.3–9.9
9	2	1	60	63	3.2%	0.4–11.0
10	0	1	85	86	0.0%	0.0–4.2
11	3	0	55	58	5.2%	0.1–14.4
12	13	3	51	67	19.4%	9.9–28.9
13	42	2	30	74	56.8%	45.5–68.0
14	50	0	24	74	67.6%	56.9–78.2
15	54	0	12	66	81.8%	72.5–91.1
16	50	1	13	64	78.1%	68.0–88.3
17	44	0	14	58	75.9%	64.8–86.9
18	57	0	8	65	87.7%	77.2–94.5
19	44	2	7	53	83.0%	70.2–91.9
20	47	1	8	56	83.9%	71.7–92.4
21	49	0	6	55	89.1%	77.7–95.9
22	49	1	9	59	83.1%	71.0–91.6
23	28	3	4	35	80.0%	63.1–91.6
24	39	0	5	44	88.6%	75.4–96.2
Total	1072	83	993	2148	49.9%	47.8–52.0

## Data Availability

Dataset available on request from the authors.

## Abbreviations

The following abbreviations are used in this manuscript:

MMR	Measles, mumps, rubella combination vaccine
MERIN	Meningitis And Encephalitis Registry of Lower Saxony and Bremen
NLGA	Public health agency of Lower Saxony
Measles IgG	Anti-measles immunoglobulin G antibodies
